# Rapid, precise quantification of bacterial cellular dimensions across a genomic-scale knockout library

**DOI:** 10.1186/s12915-017-0348-8

**Published:** 2017-02-21

**Authors:** Tristan Ursell, Timothy K. Lee, Daisuke Shiomi, Handuo Shi, Carolina Tropini, Russell D. Monds, Alexandre Colavin, Gabriel Billings, Ilina Bhaya-Grossman, Michael Broxton, Bevan Emma Huang, Hironori Niki, Kerwyn Casey Huang

**Affiliations:** 1 Department of Bioengineering, Stanford University, Stanford, CA 94305 USA; 2 Department of Physics, University of Oregon, Eugene, OR 97403 USA; 3 Biophysics Program, Stanford University School of Medicine, Stanford, CA 94305 USA; 4 National Institute of Genetics, Shizuoka, Japan; 5 Current address: Department of Life Science, Rikkyo University, Tokyo, Japan; 6 Department of Physics, Stanford University, Stanford, CA 94305 USA; 7 Department of Computer Science, Stanford University, Stanford, CA 94305 USA; 8 Janssen Research and Development, Menlo Park, CA 94025 USA; 9 Department of Microbiology and Immunology, Stanford University School of Medicine, Stanford, CA 94305 USA; 10 Current address: Synthetic Genomics Inc., La Jolla, CA 92037 USA

**Keywords:** Microbiology, Cell biology, Cell morphology, Cell shape, Imaging, Chemical genomics, Principal component analysis, Segmentation, Microscopy, Computer vision

## Abstract

**Background:**

The determination and regulation of cell morphology are critical components of cell-cycle control, fitness, and development in both single-cell and multicellular organisms. Understanding how environmental factors, chemical perturbations, and genetic differences affect cell morphology requires precise, unbiased, and validated measurements of cell-shape features.

**Results:**

Here we introduce two software packages, *Morphometrics* and *BlurLab*, that together enable automated, computationally efficient, unbiased identification of cells and morphological features. We applied these tools to bacterial cells because the small size of these cells and the subtlety of certain morphological changes have thus far obscured correlations between bacterial morphology and genotype. We used an online resource of images of the Keio knockout library of nonessential genes in the Gram-negative bacterium *Escherichia coli* to demonstrate that cell width, width variability, and length significantly correlate with each other and with drug treatments, nutrient changes, and environmental conditions. Further, we combined morphological classification of genetic variants with genetic meta-analysis to reveal novel connections among gene function, fitness, and cell morphology, thus suggesting potential functions for unknown genes and differences in modes of action of antibiotics.

**Conclusions:**

*Morphometrics* and *BlurLab* set the stage for future quantitative studies of bacterial cell shape and intracellular localization. The previously unappreciated connections between morphological parameters measured with these software packages and the cellular environment point toward novel mechanistic connections among physiological perturbations, cell fitness, and growth.

**Electronic supplementary material:**

The online version of this article (doi:10.1186/s12915-017-0348-8) contains supplementary material, which is available to authorized users.

## Background

Cell shape varies widely across bacterial species and has been linked to a diverse range of processes including adhesion, motility, pathogenicity, and differentiation [1]. The cell wall, a polymer network of sugar strands crosslinked by short peptides, is both necessary and sufficient for defining a particular cell shape [2]. The precise morphology and size of a cell is determined by the equilibrium between cell-wall mechanics and the turgor pressure caused by the high concentration of osmolytes inside the cell. Cell shape is maintained via feedback between the spatial pattern of cell-wall synthesis and the cell’s current geometry. In many rod-shaped organisms, especially those that grow by inserting cell wall material along the cylindrical region of the cell, the spatial pattern of growth is dictated by the cytoskeletal protein MreB [3], an actin homolog that forms filaments bound to the inner membrane [4]. Cell volume is positively linked with fitness [5] and increases exponentially with growth rate on different nutrient sources that vary growth rate over a wide range [6]. Moreover, in long-term evolution experiments with *Escherichia coli*, cell volume more than doubled in all evolved lines after 10,000 generations [7]. Thus, an outstanding challenge in biology is to understand the mapping between genotype and morphological phenotypes.

To achieve such an understanding requires accurate quantification of cell morphology, particularly for very subtle changes such as the sub-micron curvature preference of MreB [3]. Several computational tools were previously developed to quantify cell shape [8] in order to investigate intracellular organization and size homeostasis. The first software to interpolate cell contours at subpixel resolution was *PSICIC* [9], which has generally been applied to precisely quantify the subcellular localization of proteins. Simulations of point spread functions and their effects were combined with diffraction-limited imaging to achieve generational tracking and superior cell-division classification using *ObjectJ* [10, 11]. Another software package, *Microbetracker*, enabled segmentation of cells within a dense population [12]; *Microbetracker* and its successor *Oufti* [13] were recently used to investigate the relationships among growth rate, elongation, and division in *E. coli* [14] and *Caulobacter crescentus* [15, 16]. For rod-shaped bacteria, most quantitative studies involving cell size have essentially studied the dynamics of cell length, since cell width is generally maintained during elongation. However, *E. coli* B/r cells that experienced a nutrient upshift from minimal to rich medium increased in cell width progressively over a few doublings [17, 18], consistent with bulk measurements linking growth rate and cell volume [6]. Further, mutations in MreB [5] and key cell-wall synthesis enzymes such as PBP2 [19] have been identified that alter cell width, and sublethal doses of antibiotics such as A22, which depolymerizes MreB, or mecillinam, which inhibits PBP2, lead to cell-width increases in a concentration-dependent manner [20]. Finally, osmotic shock subtly alters cell width [21], signifying a change in turgor pressure. These data are evidence that the cell’s ability to determine its width may be important for its regulation of cell growth and fitness. While powerful for many applications, packages such as *PSICIC*, *Microbetracker*, and *MicrobeJ* [22], the latter of which has an elegant interface for tracking lineages and measuring sub-cellular localization [22–24], require a relatively large number of parameters; measurements of cell width are sensitive to the values of these parameters. Critically, our ability to link these subtle shape changes to underlying genotypes and chemical environments relies on accurate, unbiased morphological characterization.

The Keio collection of single, nonessential gene deletions in *E. coli* BW25113 is a powerful resource for discovering the phenotypes of genes of unknown function [25]. A visual screen of the qualitative shapes of the knockouts in this collection revealed only one mutant that was obviously non-rod-shaped [26]. ∆*rodZ* cells are round, and it was subsequently found that RodZ interacts with MreB [26–28]. By profiling mutants from the Keio collection across hundreds of chemical treatments and environmental conditions, the functions of several genes have been discovered [29], such as the lipoprotein co-factors LpoA/B that activate the bifunctional penicillin binding proteins PBP1A/B, respectively [30]. This chemical-genomics approach can be used to cluster genes whose functions are related by virtue of a common pathway. Given previous discoveries of close connections between cell size and growth rate [6] and size and fitness [5], measuring cell shape and size in distinct environments will likely reveal the mechanisms of growth regulation. Moreover, imaging data may constitute a phenotype vector for individual cells or populations of cells containing multiple morphological features such as cell width and length, curvature, and polar morphology [31]. A preliminary analysis of cell shape classified mutants in the Keio collection as short, normal, long, or very long (https://shigen.nig.ac.jp/ecoli/strain/resource/keioCollection/list). However, detailed features such as cell width, size variability, or polar morphology have been difficult to accurately measure due to computational and software limitations.

To quantify various aspects of cell morphology, a software platform must accurately and robustly identify changes in cell width and curvature, ideally with high computational efficiency on imaging datasets from large libraries of strains. The focus of many existing software packages has been on defining a cell contour that can be used for comparing intracellular localization patterns or for computing the dynamics of a global parameter such as cell length. Datasets estimating local cell geometry with high accuracy can enable machine-learning tools to identify low-dimensional representations of cell shape and may reveal novel biological principles connecting cell shape to other behaviors. Principal Component Analysis (PCA) was previously harnessed to analyze the cell contours of populations of cells, leading to the identification of cell-shape modes in the bacterium *C. crescentus* [32] and in keratocytes [33]. For *C. crescentus*, PCA enabled the clustering of MreB mutants [34], while for keratocytes, distinct PCA modes were strongly correlated with motility characteristics such as speed or turning [33]. In previous studies, we developed software tools to analyze cell shape in a variety of contexts. Using phase-contrast images, we measured the changes in cell width and length resulting from point mutations in MreB [5]; we verified that the changes in cell width correlated with the distance between peaks in fluorescence of a membrane dye on opposite sides of the cell [5]. In another study, we previously measured the curvature of *E. coli* cell contours and showed that MreB localized to concave regions of the cell [3], targeting new cell-wall growth to these locations and straightening the cell. When we measured the correlations among cell size, cytoskeletal dynamics, and cell twisting for cells with a range of sizes generated via genetic or chemical perturbations, we found that cells systematically altered cell-wall structure as cell width increased [20]. In a morphological screen of the effects of depleting essential genes in *B. subtilis*, partial depletion led to cell-width outliers that highlighted both shape actuators (involved in cell-wall synthesis) and modulators (e.g., involved in DNA replication) [35]. However, the molecular mechanisms that regulate cell size are currently relatively unknown, motivating an unbiased examination of a genomic-scale library.

Here we present cell-shape measurement tools in a user-friendly, computationally efficient MATLAB-based package called *Morphometrics*. This software segments cell contours from phase contrast images, fluorescence labeling of the cell surface, or cytoplasmic fluorescence, without assumptions about cell shape or size. Once cells have been identified, cell contours are determined via a straightforward, essentially parameter-free algorithm that yields robust measurements of cellular dimensions and contour curvature, allowing for automatic characterization of mutants with subtle variations in morphology. Since judging the accuracy of contour detection requires the ability to measure cell shape from images of cells with known size, we also present a separate software platform, *BlurLab*, that generates simulated fluorescence microscopy images [36]. While other software tools have been developed to generate fluorescence images for predefined shapes and structures [37], *BlurLab* addresses arbitrary distributions of fluorescent molecules in space and time and has the capacity to mimic a wide variety of techniques, features, and sources of noise in light microscopy. In the current investigation, we used *Morphometrics* to measure cell size from phase contrast and fluorescence images, and *BlurLab* to validate our measurements and to determine the relative shifts in cellular dimensions between imaging modalities. We then applied *Morphometrics* to quantify cell shape and size across ~14,000 images of the Keio collection, revealing an inverse correlation between cell width and the robustness of cell-shape maintenance. Finally, we demonstrated that cell-shape parameters such as width and length correlate with particular chemical sensitivities. Ultimately, we envision that *Morphometrics* and *Blurlab* will provide fast, reproducible quantification of cell shape as well as the ability to test quantitative models, thus complementing canonical tools for biochemistry and cell biology.

## Implementation and Results

### Assessment of the consistency of cell contours determined by different imaging modalities

To facilitate quantitative analysis of contours from cells with a wide variety of shapes and sizes, we sought to implement an algorithm that made no assumptions about specific cell shapes and that extracted a parameter-free contour not subject to user biases. The *Morphometrics* algorithm can be conceptualized in two stages. First, with a small number of user-defined parameters, discrete ‘objects’ are detected as contiguous groups of pixels through watershed and distance-transform segmentation. Second, a smooth parameter-free contour defining the boundary of each object is calculated by treating the image intensity as a metric surface on which contour ‘energy’ can be minimized, with the segmented object boundary as an initial contour guess. Further information about the algorithm can be found in the *Morphometrics* user manual included with the software download. Three types of images can be used for contour detection: i) phase contrast images, in which the cell interior appears dark; ii) interior fluorescence images, e.g. from uniformly distributed cytoplasmic fluorescent proteins; and iii) peripheral fluorescence images, e.g. from membrane dye. Calculating the magnitude of the image gradient transforms the first two image types into an intensity map similar to that given by a fluorescence marker on the surface, which constitutes the common basis for calculating contours.

After optional image scaling, contrast adjustment, and background removal, the software offers multiple algorithms for segmenting contiguous groups of pixels (objects) that meet specified constraints on size and intensity. Each object is checked against criteria for false-positive detection based on the ratio of interior to boundary pixel intensities and, depending on user input, these objects may be linked with objects in other frames for tracking across a set of time-lapse images (see examples in the *Morphometrics* user manual). Parameters may be tested on individual images before being applied to the processing of large data sets. Ultimately, each segmented region serves as a seed to begin contour fitting. Once a contiguous object composed of discrete pixels is identified, a contour is calculated by treating the intensity features of the object as an interpolated energy landscape and then fitting a closed-loop contour in continuous coordinates to the minima of that energy landscape. From the cell contours, *Morphometrics* can be used to calculate an interior mesh; for rod-shaped cells, this mesh defines a cellular coordinate system with a midline and associated perpendicular meshlines that connect the two sides of the cell, thereby also measuring local cell width along the midline. The contour is also used to calculate one-dimensional profiles such as curvature (a measure of the radius of the circle that best fits the contour surrounding a particular point and whether the contour is concave or convex), fluorescence signals along the cell boundary (e.g., membrane dye, surface markers [3], or membrane-bound proteins [38]), or fluorescence signals along the interior centerline of the cell, among other features. Optical shifts between fluorescence imaging channels can be corrected by translating the contour coordinates by an amount appropriate to the particular imaging system; these translation values can be determined visually using the included post-processing contour viewing software.

**Fig. 1:**
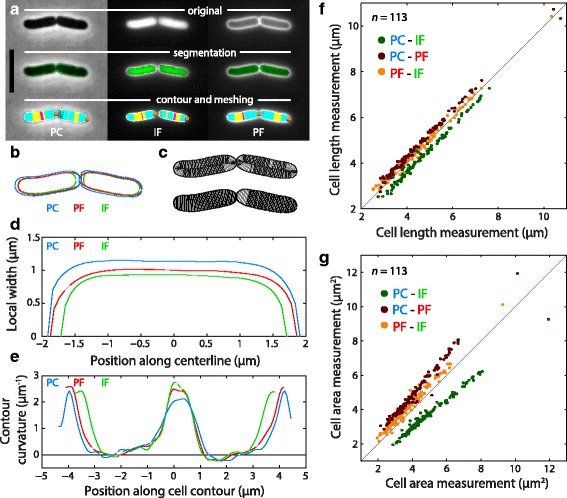
Quantitation demonstrates consistency among contour measurements from different imaging modalities, despite small differences. **a** *E. coli* cells imaged using phase contrast (PC), interior fluorescence (IF) from cytoplasmic GFP, and peripheral fluorescence (PF) from the surface marker Alexa 594-Wheat Germ Agglutinin. *Top*: original images; middle: segmentation output from *Morphometrics*; *bottom*: extracted contours and meshlines. The pole from which all contours are measured is marked by *orange* and *maroon* dots for the beginning and end of the contours, respectively. Meshlines are colored cyan if the cell contour has positive (*outward*) curvature at both endpoints, maroon if the contour has negative curvature (inward) at both endpoints, and yellow if the contour has opposite signs of curvature (indicating a region where the cell body curves). Scale bar: 5 μm. **b** Overlay of the PC, IF, and PF cell outlines from (**a**). **c** Branching mesh (*top*) and centerline mesh (*bottom*) of the cells in (**a**), using the PF contours. These meshes are used to measure width profiles along the cell. **d** Comparison of the single-cell width profiles from pole to pole among all three imaging modalities for the cell on the left in (**a**). PC contours consistently estimate larger widths than PF or IF contours. **e** The PC, IF, and PF contours have similar curvature profiles, with slight differences consistent with width differences in (**d**). **f** Differences in cell-length measurements among imaging modalities are consistent across a wide range of cell lengths. In the legend, the first and second modality for each color correspond to the measurements along the *y*- and *x*-axes, respectively. Black line is *y* = *x*. **g** In cell area measurements, differences in length between imaging modalities shown in (**f**) are exacerbated due to width-dependent offsets in cell widths between imaging modalities, as shown in (**d**)

To illustrate the capabilities of *Morphometrics* for contour detection, we stained *E. coli* cells expressing cytoplasmic GFP with the surface marker Alexa 594-conjugated Wheat Germ Agglutinin [3] and imaged the cells using phase contrast and epifluorescence microscopy (Fig. 1a, top). *Morphometrics* successfully segmented isolated and dividing cells (Fig. 1a, middle) from all three imaging modalities (phase contrast (PC), interior fluorescence (IF), and peripheral fluorescence (PF)), leading to three sets of contours, with the PF contour exterior to the IF contour as expected (Fig. 1b). The meshlines for these cells (Fig. 1c) define a cellular grid that can be associated with regions of the contour or cell midline that show positive or negative curvature (Fig. 1a, bottom).

From these cells and their associated meshline grids, we measured width profiles along the midline, obtaining values that increased from 0 at the two poles to ~1 μm near the middle of the cell (Fig. 1d). As previously reported [39], there was little intracellular variability in cell width along the midline away from the poles, and all three profiles showed similar variability (Fig. 1d). The contour measurements represented an approximately cylindrical body with hemispherical endcaps (Fig. 1d), although all three contour measurements displayed slight variations in curvature (Fig. 1e) that were previously shown to correlate with MreB localization [3, 40].

**Fig. 2:**
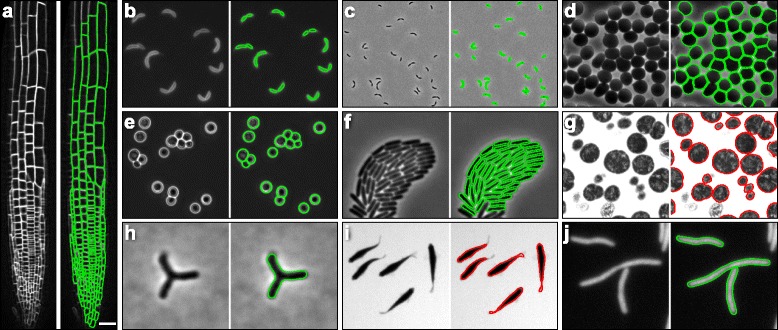
*Morphometrics* achieves unbiased contour extraction across a wide range of cell shapes and object types, including densely packed communities and tissues. **a** Contours extracted from the root-tip cells of an *A. thaliana* plant expressing a YFP-fusion to the membrane protein LTI6B, showing that *Morphometrics* is capable of segmentation of a complex tissue. Scale bar: 15 μm. **b**, **c** Contours extracted in PF mode (**b**) or PC mode (**c**) from FM4-64-labeled *C. crescentus* cells. **d** Contours extracted in PC from approximately spherical hypotonic red blood cells. **e** Contours extracted in PC from the budding yeast *S. cerevisiae*. **f** Contours extracted in PC from a densely packed *P. aeruginosa* community. **g** Contours extracted from transmission electron microscopy images of *N. gonorrhoeae*. **h** Contours extracted in PC from branched *B. bifidum* DSM20213 cells. **i** Contours extracted from bright-field microscopy of live zebrafish *Danio rerio*. **j** Contours extracted in IF from filamentous *E. coli* expressing cytoplasmic GFP

PC, IF, and PF images are expected to provide distinct positions of cell boundaries. Across many cells, there were strong correlations among length measurements from the three modalities; lengths from PC images were consistently larger than those from IF or PF images by ~400 nm (Fig. 1f). Similar comparative behavior was observed in cell width measurements (Additional file 1: Figure S1). The combined effects of shifts in width (Fig. 1d, Additional file 1: Figure S1) and length (Fig. 1f) led to an increasing divergence in the area measurements of the three imaging modalities across many cells (Fig. 1g). Nonetheless, all three imaging modalities were highly correlated, indicating a consistent picture of cellular dimensions that can be applied to wide variety of organisms. To demonstrate the utility of *Morphometrics* for unbiased contour detection across a wide range of object shapes, we analyzed PF images of the root tissue of *Arabidopsis thaliana* plants (Fig. 2a), PF (Fig. 2b) and PC images (Fig. 2c) of curved rod-like *Caulobacter crescentus,* PC images of red blood cells (Fig. 2d), brightfield images of budding yeast *Saccharomyces cerevisiae* (Fig. 2e), PC images of dense *Pseudomonas aeruginosa* communities (Fig. 2f), transmission electron microscopy images of *Neisseria gonorrhoeae* (Fig. 2g), PC images of branched *Bifidobacterium breve* DSM20213 (Fig. 2h), brightfield images of zebrafish (Fig. 2i), and IF images of filamentous *E. coli* (Fig. 2j). In all cases, *Morphometrics* successfully segmented the cells or organisms, regardless of shape or imaging modality.

### Simulated fluorescence microscopy for validation of quantitative analyses

Given the differences in cellular dimensions extracted from PC, IF, and PF data (Fig. 1), we wondered which modality accurately represented cell size. Other experimental methodologies with higher resolution such as electron microscopy still do not provide a “true” measure of cell size, since sample preparation likely perturbs the cell, for example by disrupting turgor pressure. To identify a strategy for comparing measurements with known geometric parameters, we developed *BlurLab*, a software package that generates simulated fluorescence images. *BlurLab* takes as input a set of locations of fluorescent molecules and convolves these locations with a point spread function (PSF) to generate a simulated image. The PSF can be directly measured for a particular microscope and objective using sub-diffraction-limited particles such as quantum dots or fluorescent beads; alternatively, *BlurLab* can generate a PSF for a given set of objective parameters (numerical aperture, wavelength, magnification, index of refraction, and pixel size). *BlurLab* can also mimic camera noise, thermal noise, and shot noise, yielding simulated images that are more realistic for head-to-head comparison with experimental images. Additional *BlurLab* functionalities include simulation of imaging at other focal planes for creating *z*-stacks, modeling total internal reflection fluorescence imaging, boxcar averaging of positions during simulated time-lapse imaging to account for particle motion during the exposure interval, simulating mean-field and stochastic photobleaching, and simulating fluorescence recovery after photobleaching. Detailed descriptions of these functionalities and examples can be found in the *BlurLab* manual.

**Fig. 3:**
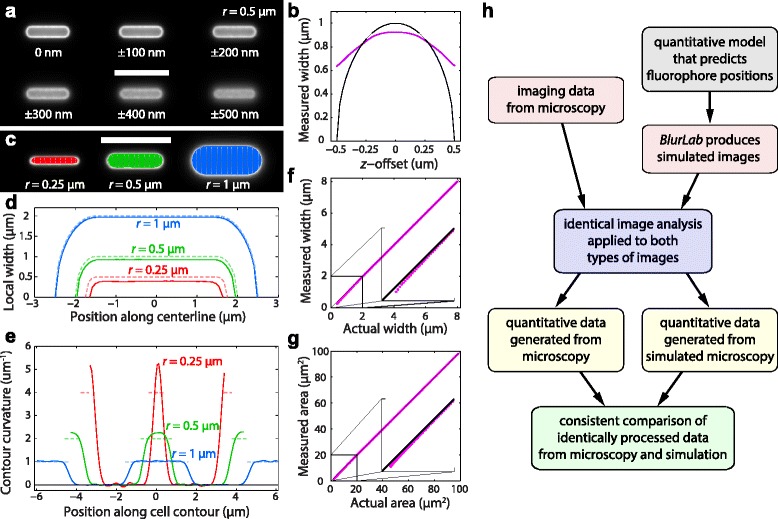
Simulated fluorescence images permit quantification of the accuracy of cell-geometry measurements. **a** Simulated fluorescence images of uniform surface labeling of an in silico cell with width 2*r* = 1 μm and length 4 μm. The focal plane of each image relative to the cell midplane is indicated. Scale bar: 5 μm. **b** The measured width (*magenta*) of the middle cell in (**a**) at different offsets relative to the midplane, does not vary as strongly as the actual width (*black*) at that focal plane. **c**-**e** *BlurLab* permits the precise quantification of errors in geometry measurements from extracted contours and meshlines (**c**), width profiles (**d**), and curvature profiles (**e**) for cells of different radii *r*. Scale bar in (**c**): 5 μm. The colors of the meshlines in (**c**) are maintained in (**d**, **e**). Dashed curves in (**d**, **e**) are the actual values for the in silico cells. (**f**, **g**) Width (**f**) and area (**g**) display a systematic bias for narrow cells relative to the lines of equal measured and actual area (*black*). This bias is negligible for cells with width above ~1.5 μm. **h** Conceptual flow chart of the utility of *BlurLab* for quantitative comparison of experimental and simulated images to test the validity of an underlying model (here, measurements of cell size and geometry)

To validate cellular dimensions and morphological features of *E. coli* cells, we used *BlurLab* to generate sets of uniformly distributed molecules at high density on the surface of cylinders with hemispherical endcaps over a range of cell widths and lengths. For an in silico cell with width 1 μm, we also simulated images of the same cell at focal planes up to 500 nm above and below the cell midplane (Fig. 3a). As the cell goes out of focus, the image attributes used for contour fitting become blurred by the PSF. Nonetheless, a bright boundary, the signature of surface-bound fluorescence, was evident in each image (Fig. 3a). We then applied *Morphometrics* to each simulated image and successfully resolved a cell contour at each *z*-offset. The width measured from these cell contours peaked at the midplane and monotonically decreased as the offset from the cell midplane increased (Fig. 3b). These data illustrate the importance of midplane focus, and provide an estimate of the deviation in width measurement when using out-of-focus cells. Interestingly, these data also show that the measured cell width (magenta line in Fig. 3b) is more robust to changes in the focal plane than the actual cell width at a given focal plane (black line in Fig. 3b).

Next, we examined the contours extracted from simulated images of in silico cells with different widths (Fig. 3c). The error in the extracted contour was practically zero for widths greater than 2 μm, but increased as cell width decreased (Fig. 3d). The extracted contours were smaller than the true midplane contours because the cell curvature introduced light from out-of-focus planes in which the cellular cross-section had a smaller width; for cell widths much larger than the wavelength of light used for imaging, this curvature became negligible (Fig. 3d). To determine the accuracy of other geometric measurements, we computed the curvature along the cell contour. The curvature along a 2-μm in silico cell was relatively accurate everywhere except in the transition region between the cylinder and the hemispherical end caps (Fig. 3e), where the PSF blurred the step-function curvature into a smooth transition. For a 1-μm in silico cell, the curvature remained accurate along the cylindrical portions of the cell (where the measured curvature was close to zero), although there was a slight overestimate of the curvature at the ends of the cell (Fig. 3e); this error was exacerbated as the cell width was further narrowed (Fig. 3e). We note that these systematic morphological errors with decreasing cell size result from the limitations of light imaging at wavelengths comparable to the cell size, not from imprecision in fluorescence simulation or contour detection.

To evaluate the overall bias, we used *BlurLab* to compute the difference between the actual and measured widths and areas for in silico cells 400 nm to 8 μm in width and a range of cell lengths in the peripheral fluorescence modality. The error in the width asymptotically approached zero, and was essentially undetectable in cells with width above ~1.5 μm (Fig. 3f). Error in area behaved in a similar fashion, regardless of cell length (Fig. 3g), indicating that PF measurements provide an accurate measure of cell length without systematic bias. This application illustrates the intended purpose and power of *BlurLab*: to assess the accuracy of imaging data in the context of a particular model by consistently comparing analyses of both experimental and simulated images (Fig. 3h).

### Morphological analysis of a genomic library of nonessential gene deletions

**Fig. 4:**
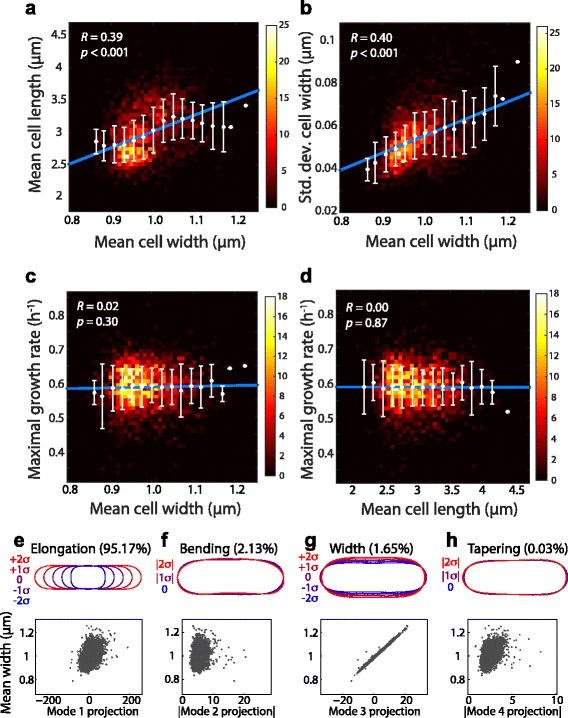
Morphological analysis of the Keio collection reveals correlations between morphological features but not with growth rate. Contours from >150 cells per Keio deletion strain were extracted from images acquired from the NBRP repository and used to compute the mean length and width and standard deviation of cell width across each population. In (**a**-**d**), white circles and error bars were obtained by binning strains by mean width or length; blue lines are the fit to binned averages. *R* is Pearson’s correlation coefficient. **a**-**b** Heatmaps of the number of strains with geometry parameters in each bin show that mean length (**a**) and standard deviation of mean width (**b**) are positively correlated with mean width. **c**-**d** Neither mean width (**c**) nor mean length (**d**) are correlated with maximal growth rate, as measured from microplate growth curves in [41]. **e**-**h** Top: Representations of the PCA modes around the mean shape. Modes 1–4 represent elongation, bending, width, and tapering, respectively. Bottom: scatter plots of the proportion of modes 1–4 and mean cell width of each strain demonstrate that width is correlated with variation represented by modes 1, 3, and 4

To demonstrate the efficacy of *Morphometrics* for rapid quantification of bacterial morphology, we analyzed ~14,000 images of the Keio collection, a collection of single knockouts of all non-essential genes in *E. coli* [25]. We obtained phase contrast images from the National BioResource Project and segmented isolated cells from each strain in an unbiased manner (Methods). Mean cell width varied from ~0.8 to 1.2 μm, and mean cell length varied from ~2.5 to 4 μm (Fig. 4a, Additional file 2: Figure S2). Interestingly, mean cell width and length were strongly correlated with each other (*R* = 0.39, Student’s *t*-test: *p* < 0.001, Fig. 4a), as were mean cell width and length standard deviation (Additional file 3: Figure S3). From our data, we determined the distributions of morphological parameters such as mean width and length across the population of cells for each strain. Moreover, we utilized the meshing of each cell to measure the local cell width (distance across the cell at each point along the contour), from which we calculated the mean variability in cell width within individual cells in the population. Both the standard deviation across the population (Fig. 4b) and the intracellular fractional width variability (Additional file 4: Figure S4) increased with mean cell width, indicating that cells are increasingly unable to maintain cell width as they widen. Interestingly, although wild-type *E. coli* cells increase in cell size with nutrient-induced increases in growth rate [6], we found no significant correlations between maximal growth rate (as determined by microplate growth curves in [41]) and cell width (Fig. 4c) or length (Fig. 4d). We note that these results are not contradictory; for example, a previous study showed that cell size is not correlated with growth rate within a population of cells [14].

To identify other morphological correlations across the library or in particular strains, we aligned 150 cell contours from each strain of the Keio collection for which at least 150 contours were available in order to calculate an average cell shape, and performed PCA on the covariance matrix to identify significant shape variations. The first two, three, and four PCA modes accounted for 97.3%, 99.0% and 99.3% of variation in cell shape, respectively (Fig. 4e–h), and by far the greatest amount of variation was accounted for by a mode that clearly captured elongation (Fig. 4e). This result, which is not surprising for rod-shaped growth, indicates that length changes are the most significant source of shape variation within the Keio library. Nonetheless, the next three modes representing cell bending, widening, and tapering, respectively (Fig. 4f–h), have potential for revealing cells or strains that are shape outliers. Mean width strongly correlated with PCA mode 3 (width, *R* = 0.998, Student’s *t*-test: *p* < 0.001) and with the projection from mode 1 (length, *R* = 0.43, Student’s *t*-test: *p* < 0.001) (Fig. 4e, bottom), as expected based on the correlation between length and width noted above (Fig. 4a). There was also a significant correlation between mean width and mode 4 (tapering, *R* = 0.49, Student’s *t*-test: *p* < 0.001) (Fig. 4h, bottom), potentially indicating a connection between cell-width determination and cell division. Our analysis demonstrates that most of the variation in cell shape is captured by length and width, although other morphological features such as tapering may be informative for characterizing certain outlier strains. PCA of the correlation matrix, which involves rescaling that avoids heavy skewing by cell length variation, led to an increased emphasis on cell width, bending, and tapering in the decomposition, with the mode corresponding to the largest eigenvalue representing tip morphology (Additional file 5: Figure S5). Taken together, correlations among features suggest underlying feedback between elongation and division in rod-shaped cells [42].

### Identification of chemical sensitivities correlated with cellular dimensions

Given the range of cellular dimensions across the Keio library and the correlations between morphological observables, we wanted to systematically probe the physiological significance of cell size. We previously found that the MreB^A53T^ mutation led to wider cells during growth in a variety of carbon sources; cells harboring this mutation had a large gain in fitness when competed against the parental strain in glucose-rich medium [5]. However, the change in fitness was carbon-source dependent, with neutral fitness in lactose and a reduction in fitness in galactose [5]. We also previously observed that sublethal treatment with the MreB inhibitor A22 led to a dose-dependent increase in cell width in wild-type *E. coli* MG1655 cells [20]. Based on these data and the increase in width variability with increasing mean cell width across the Keio collection detected here (Fig. 4c), we hypothesized that wider cells may be more sensitive to A22 than thinner cells, and more generally that morphological observables may be predictive of the severity of phenotypes in certain environments or chemical treatments.

**Fig. 5:**
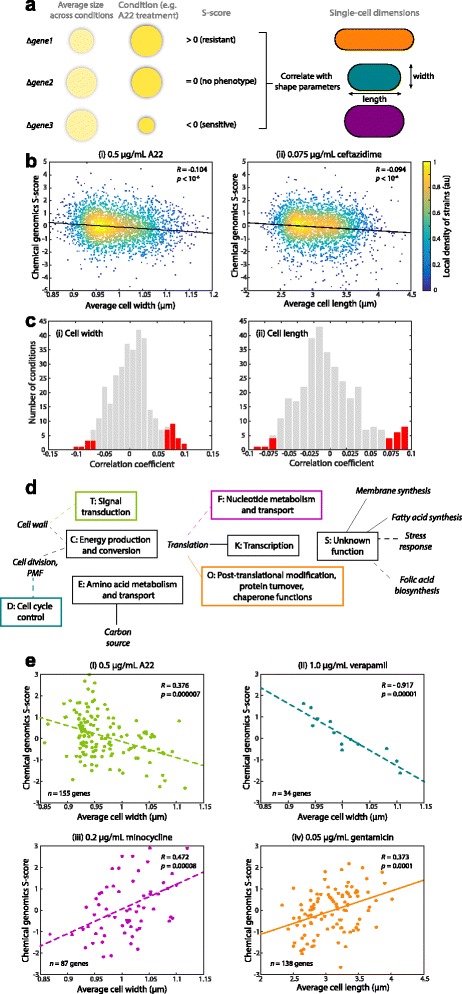
Morphological parameters predict certain chemical sensitivities. (**a**) Schematic of S-score interpretation and strategy for correlating with cell-shape parameters. Gene-condition pairs that result in bigger (smaller) colonies, normalized to the size of a wild-type colony in the same condition, than expected based on average size across all conditions have positive (negative) S-scores. (**b**) (*i*) After 0.5 μg/mL A22 treatment, S-scores of Keio-collection knockouts were negatively correlated with mean cell width across populations of cells of each strain, indicating that wider strains are generally more sensitive to A22. (*ii*) S-scores after treatment with 0.075 μg/mL ceftazidime, a cephalosporin division inhibitor, were negatively correlated with cell length, indicating that longer cells are more sensitive to ceftazidime. *p*-values computed with Student’s t-test. (**c**) The distribution of correlation coefficients with (*i*) mean cell width and (*ii*) length across the 324 conditions screened in [29]. *Red* bars highlight conditions with statistically significant correlations, Bonferroni corrected for multiple hypothesis testing. (**d**) Connections between Clusters of Orthologous Groups (COGs) and drugs that target particular processes. A connection is defined by a statistically significant correlation between cell width (*dashed lines*) or length (*solid lines*) and S-scores for knockouts of genes within the COG class indicated in the rectangles. (**e**) Examples of COG-specific correlations, with the colors of the dots and best-fit line the same as the appropriate COG rectangle in (**d**). *p*-values computed with Student’s *t*-test. (i) Sensitivity to 0.5 μg/mL A22 is negatively correlated with cell width in knockouts of genes related to signal transduction. (*ii*) Sensitivity to 1.0 μg/mL verapamil (calcium channel blocker) is negatively correlated with cell width in knockouts of genes related to the cell cycle. (*iii*) Sensitivity to 0.2 μg/mL minocycline (protein synthesis inhibitor) is positively correlated with cell width in knockouts of genes related to nucleotide metabolism and transport. (*iv*) Sensitivity to 0.05 μg/mL gentamycin (protein synthesis inhibitor) is positively correlated with cell length in knockouts of genes related to post-translational modification, protein turnover, chaperone functions

To test these hypotheses, we made use of an existing chemical genomics dataset [29] in which the Keio collection was grown as colonies on agar plates in 324 conditions including media, drugs, dyes, detergents, metal stresses, and hormones. From the colony sizes, a statistic called an S-score was previously computed to represent the severity of the growth phenotype in each condition [29]; a positive/negative S-score indicates more/less growth than expected based on the changes to wildtype in the condition of interest (Fig. 5a). We compared our quantification of cellular dimensions with the previously reported S-scores from treatment with 0.5 μg/mL A22 for each knockout, and detected a significant correlation between cell width and S-score (Fig. 5b, Pearson correlation coefficient *R* = −0.10, Student’s *t*-test: *p* < 10^−6^), consistent with our hypothesis. We then wondered whether other chemical or environmental perturbations were correlated with mean cell width or length. We calculated the correlation coefficients of these quantities with each of the 324 condition datasets (Fig. 5c) and determined statistical significance with a Bonferroni correction for multiple hypothesis testing (Methods). For cell width, A22 treatment was the most significant negative correlate; six other compounds (ignoring differences in concentration) also exhibited significant negative correlation (Additional file 6: Table S1) [29]. Of these compounds, three also targeted cell-wall synthesis (the β-lactam cefaclor, the peptide bacitracin, and the amino acid derivative D-cycloserine), possibly indicating inhibitory effects similar to those of A22. The list also included compounds targeting the membrane or proton motive force (the Ca^2+^-channel inhibitor verapamil and the detergent taurocholate) and translation (50S inhibitor erthyromycin) (Fig. 5d), indicating potential links between cell-width control and other metabolic processes.

There were 15 unique conditions for which the knockouts had S-scores with significant positive correlation with cell width (Additional file 6: Table S2) [29], indicating that wider cells were less sensitive. These conditions target a broader range of cellular processes, including DNA/RNA, stress, and fatty acid metabolism in addition to the ribosome and cell-wall and membrane synthesis (Additional file 6: Table S2) [29]. Carbonyl cyanide m-chlorophenyl hydrazone, a proton ionophore that inhibits oxidative phosphorylation, was positively correlated at all tested concentrations (Additional file 6: Table S2) [29]; interestingly, we observed in a separate study that this ionophore increases bending rigidity, which is an expected outcome of increased cell width based on the mechanics of a thin cylindrical shell. There was also a compound (theophylline) with an unknown target that exhibited a significant positive correlation with cell width (Additional file 6: Table S2) [29], indicating that cell-size profiling may be an effective tool for associating chemical exposure with the molecular pathways that control cell morphology.

We next considered correlations of S-scores with cell length. Six conditions were associated with significant negative correlations (Fig. 5cii): high iron, treatment with the cell-wall inhibitor ceftazidime, and four carbon-source limitations, which may reflect the known coupling of cell size with nutrient-dependent growth rate [6]. Of the eight conditions positively correlated with length (Fig. 5cii), several involved ribosomal inhibitors (*n* = 3), inhibitors of fatty acid and membrane synthesis (*n* = 2), or DNA/RNA synthesis (*n* = 1). In some cases, correlations indicated differences in the underlying mode of drug action; for example, the cephalosporin cefaclor was positively correlated with cell length, while the structurally similar compound ceftazidime was negatively correlated with length.

Given that the observed correlations between cell size and S-scores involved chemicals with many target processes, we conjectured that the correlations for subsets of strains deleted for genes with similar functions may yield further insight into the relationships between cell size and cellular processes. We separated genes into 23 Clusters of Orthologous Groups, a common classification scheme [43]. As compared to the correlations from the full library (Fig. 5c), we detected higher correlations between cell size and S-scores within some of these gene clusters (Fig. 5d,e; Additional file 6: Table S3) [29]. A22 again was significantly (Student’s *t*-test) and negatively correlated with cell width for strains harboring deletions of genes associated with signal transduction mechanisms (Fig. 5ei), indicating that signaling pathways, such as those activated in response to stress, may respond to the changes in cell width caused by A22 treatment. Although the COG for cell-cycle control, cell division, and chromosome partitioning comprises only 34 genes, sensitivity to the division inhibitor verapamil nevertheless exhibited a strong and significant correlation of −0.92 with cell width (Fig. 5eii), while S-scores for minocycline sensitivity of knockouts of genes involved in nucleotide metabolism and transport were positivity correlated with cell width (*R* = 0.47, Fig. 5eiii). Surprisingly, for the large number of strains carrying deletions of genes of unknown function (COG class S, *n* = 918 genes), mean cell width was negatively correlated with sensitivity to low iron stress and the folic acid synthesis inhibitor sulfamethizole (Fig. 5d). Strains harboring deletions in genes involved in amino-acid transport and metabolism had mean cell lengths that were negatively correlated with sensitivity to four carbon-source starvations (Fig. 5d), while cells harboring deletions in genes involved in transcription and post-translational modification, protein turnover, and chaperones had lengths positively correlated with ribosomal inhibitor sensitivity (Fig. 5eiv). As with cell width, strains deleted for genes of unknown function had lengths both positively and negatively correlated with some treatments (Additional file 6: Table S4) [29], further supporting the importance of these genes to cell shape. Taken together, our correlation analyses based on the entire Keio collection and subdivided into Clusters of Orthologous Groups suggest that a variety of intracellular factors, beyond those controlling cell-wall synthesis or turgor, contribute to the cell’s determination of its size, and that responses to some extracellular perturbations have general connections with cell size (Fig. 5d). These correlations were revealed through precise and automated measurements of cellular dimensions that were enabled by our open-source software packages *Morphometrics* and *BlurLab.*

## Discussion and Conclusions

Quantifying subtle connections among environmental conditions, cellular morphology, and genetics is a crucial step in uncovering new biological roles for genes and their corresponding phenotypes. Microscopy is a powerful workhorse for establishing these connections, but the spatial constraints of light microscopy and our computational assumptions limit the set of measurable morphological perturbations. We developed *Morphometrics* and *BlurLab* to expand the utility of multiple imaging modalities and to minimize the presence of bias in image-based analyses. Here, we applied our software to bacteria, which are a technically challenging class of organisms to explore with quantitative image processing due to their small size and strong connections among fitness, genotype, and morphology. We envision that combining the morphological measurement techniques of *Morphometrics* with the unbiased hypothesis testing of *BlurLab* will enable the quantitative and automated characterization of libraries targeting cell morphology (such as libraries constructed through error-prone PCR mutagenesis of genes that affect cell morphology [5] and the set of Keio mutants with a range of cell lengths and widths (Fig. 4)) as well as the growing collection of genomic libraries, including knockout libraries of *Salmonella typhimurium* [44] and transposon-based libraries of the pathogen *Pseudomonas aeruginosa* [45] and the gut commensal *Bacteroides thetaiotaomicron* [46]. The relationships we discovered among cell length, cell width, and width variability (Fig. 4) across the genomic-scale Keio collection suggest general links between cellular physiology and cell size [42]. Screens that used the Keio collection to reveal new phenotypes previously focused on growth [29] or envelope permeability [47], both of which naturally led to studies of cell shape. Since *Morphometrics* is sensitive to small morphological variations and can rapidly analyze tens of thousands of images, it crucially enables forward genetic screens for genes or mutations that affect a morphological phenotype.

The relatively high throughput and unbiased morphological characterization of *Morphometrics* make it well-suited to the study of morphological and functional connections in genomic libraries, to the screening of environmental conditions like carbon sources, osmolytes, and antibiotics, and to analyses of dynamic cell-cycle data via time-lapse imaging [48]. Similarly, new rules of cellular homeostasis encoded by time-dependent morphology in many organisms [14–16, 49] will be accessible with our software, particularly when spatial resolution is limited. It remains to be seen to what extent detailed measurements of cell morphology will be sufficient to uncover relationships among genotypes, chemical treatments, and cell morphology (Fig. 5); in some cases, lack of shape variation or degeneracies in shape phenotypes may make identification of a morphological signature challenging. For instance, drug treatments and attendant mutations could lead to global changes in gene expression such as a stress response that result in similar non-specific changes in cell morphology (e.g. filamentation).

While we have focused on bacteria, *Morphometrics* is amenable to morphological analysis of any organism for which high-contrast images can be obtained, especially walled organisms like plants (Fig. 2a) [50] and fungi (Fig. 2e) [51]. *Morphometrics* has relatively few parameters and makes no assumptions about object morphology, strengths that are important considerations when tuning algorithms to examine new organisms with poorly understood morphology and growth cycles and when developing custom scripts for post-processing, such as the detection of spatial and temporal morphological correlations [3], detailed localization studies [3], and testing of biophysical models [39]. The small size of bacteria leads to a smaller visual dynamic range, which makes human observers more prone to apophenia (seeing meaningful patterns in random data). *BlurLab* has a wide array of potential applications in defining null hypotheses about fluorescence imaging data, such as the expected distribution of a uniform surface label at the poles versus the lateral walls of a rod-shaped cell, the integrated intensity of a homogeneous cytoplasmic label such as 4′,6-diamidino-2-phenylindole (DAPI) as a function of cell size (given that wider cells have a greater fraction of the cell volume at the edges of the focal plane), and the level of noise in cytoplasmic distributions as a function of molecule number for testing whether noise is spatially heterogeneous. *BlurLab* can be coupled to any analysis platform, and should have growing utility given recent genome-scale screens of fluorescence localization [48, 52]. We note that evaluations of null hypotheses should also be important for eukaryotic cells, for which it is equally critical to connect molecular models to diffraction-limited fluorescence images [53].

For large data sets, image processing must be efficient in terms of computational resources and speed. The data structures, algorithms, and graphical user interfaces of *Morphometrics* and *Blurlab* are designed to exploit optimized image processing algorithms in MATLAB and to allow users to easily construct seamless custom scripts for data analysis [3]; these packages also take advantage of the large body of user-generated algorithms online (e.g. the MATLAB File Exchange). Additionally, high-level programming languages reduce the barrier to acquire coding skills and ensure that the varied and general needs of the quantitative imaging community for custom analysis can be met. Speed limitations in MATLAB can be resolved by recoding computationally intensive subroutines in C, as we did in *Morphometrics*. While *Morphometrics* and *BlurLab* enable new levels of precision and quantitative morphological characterization, like any analysis software they have certain limitations. Ultimately, the precision of contours from *Morphometrics* is limited by i) image quality, specifically spatial resolution, signal-to-noise, evenness of illumination, dynamic range of intensities, and degree of saturation; and ii) sample characteristics such as object contrast, the proximity of objects and attendant overlap in the light fields, the degree of blurring due to motion, and, where fine segmentation is desired, the number and degree of construction points in the image outline. As a general rule of thumb, images that are visually difficult to segment will be difficult for *Morphometrics* to segment. *BlurLab* is a simulator for linear optical microscopy, but specific nuances of an optical system or objective, atypical or time-dependent noise sources, camera chip-specific noise, and sample-dependent effects (e.g. absorption, quenching, or a sample’s index of refraction) all reduce the accuracy of simulation. Similar limitations will very likely apply to *any* image analysis or simulation software.

The importance of quantitation in cell biology will continue to increase, and the small size of bacteria and lack of organelles make both *Morphometrics* and *BlurLab* especially important for identifying subtle localization and morphological phenotypes in these organisms. However, despite the breadth and versatility of *Morphometrics* and *BlurLab*, significant challenges for computational image processing remain, including reconstruction of three-dimensional morphology in both static and dynamic environments, segmentation and tracking of dense and/or highly dynamic groups of objects, and the development of algorithms to process different imaging modalities such as fluorescence recovery after photobleaching, total internal reflection fluorescence, and super-resolution imaging. *Morphometrics* and *BlurLab* should serve as a foundation for developing new software to address these challenges.

Precise quantification of cell morphology in bacteria and eukaryotes will undoubtedly be a valuable tool for mapping genotype-phenotype relationships. Extending the analysis of static images carried out here to the dynamic response of cells to perturbations, for instance in microfluidic chambers, can further reveal the physiological basis of a particular phenotype, such as the mechanism of cell death during entry into stationary phase in an *E. coli* mutant with disrupted lipid homeostasis [54]. Excitingly, cell morphology can even serve as a diagnostic tool for assessing cellular states in diseases such as cancer. To fully exploit the information obtained through these studies, we must develop and implement computational tools with high levels of accuracy and precision and couple them to methods for validation and compelling visual display. As demonstrated here, *Morphometrics* and *BlurLab* constitute an important step toward meeting these goals.

## Methods

### Single-cell imaging

Wild-type MG1655 *E. coli* cells expressing cytoplasmic GFP and a kanamycin resistance cassette from plasmid pZS21-GFP (gift from Tom Silhavy, Princeton University) were labeled with the N-acetylglucosamine- and sialic acid-specific lectin Wheat Germ Agglutinin conjugated to Alexa-594 (Life Technologies). Five milliliters of cells were grown in lysogeny broth (LB) with shaking at 37 °C to exponential phase (optical density at 620 nm ∼ 0.5). One milliliter of cells was washed with fresh LB via centrifugation (10,000 g) and resuspension in 1 mL of LB and subsequently diluted 1:10 into 1 mL of fresh LB. Twenty-five microliters of a once-frozen 1 mg/mL fluorescent Wheat Germ Agglutinin stock solution were added, and the sample was briefly vortexed. Cells were incubated with the lectin for 20 min (approximately one cell cycle) with shaking at 37 °C in the dark. After incubation, cells were washed twice with fresh LB to remove excess lectin, and 5 μL of labeled cells were deposited onto a LB + 1% agarose pad, allowed to air dry on the pad, and promptly sealed with a #1.5 coverslip in a 125-μL FastWell (Grace BioLabs).

Labeled cells were imaged on a Nikon Eclipse Ti-E inverted fluorescence microscope with a 100X (NA 1.40) oil-immersion objective (Nikon Instruments). Images were collected using an Andor DU885 EMCCD camera (Andor Technology). Cells were maintained at 37 °C during imaging with an active-control environmental chamber (HaisonTech). Images were collected using μManager v. 1.3 [55].

### Imaging of the Keio collection

Images were obtained from the NBRP. In brief, to obtain these images, strains from the Keio collection were inoculated in LB with 30 μg/mL kanamycin and grown overnight in 96-well plates at 30 °C. Cells were then diluted in LB plus 30 μg/mL kanamycin and grown for 2 h at 37 °C. After reaching exponential phase, cells were harvested via centrifugation and resuspended in LB. These cells were mounted on poly-lysine-coated cover-slips, fixed with methanol, washed with water, and stained with (4′,6-diamidino-2-phenylindole).

### Analysis of Keio images

Keio collection images from the NBRP (1–3 per strain) were analyzed using *Morphometrics*. Cells with segmentation errors were filtered by only including contours with two identifiable points of high curvature (corresponding to the poles). This filtering eliminated segmentation errors, which we evaluated with manual curation (data not shown). Cell length and width were calculated according to a mesh representation of the cell contour computed by *Morphometrics*. Subsequent analyses only included strains for which 150 cells passed the above filtering step (2465/4353 strains).

### PCA

Shape-variation modes were calculated from cell contours using PCA. Briefly, the center of mass and principal axes were calculated from the cell contour and the coordinates were shifted and rotated to a common alignment. Then, 300 equally spaced points were sampled from the cell contour using linear interpolation. A mean cell contour was subtracted from each contour and principal components were calculated using the eigenvalue decomposition of the covariance matrix between contour coordinates.

### Statistical analyses

For comparing cell morphology to chemical sensitivity (Fig. 5), Pearson’s correlation coefficient between the mean cell width or length against the S-score across all strains was calculated for each chemical condition. The statistical significance of each correlation was calculated with a Student’s *t*-distribution, Bonferroni-corrected with the number of conditions.

## Additional files

Additional file 1: Figure S1. Width measurements were similarly consistent as length and area across imaging modalities, despite small differences. (A) Differences in width measurements among imaging modalities displayed the same behaviors as length (Fig. 1f) and area (Fig. 1g) across a wide range of cell widths. In the legend, the first and second modality for each color correspond to the measurements along the *y*- and *x*-axes, respectively. Black line is *y* = *x*. (B) Histograms of cell widths in (A) as measured by each imaging modality. (PDF 132 kb)
Additional file 2: Figure S2. For all strains used for PCA, the analyzed population had a broad distribution of cell lengths. (PDF 412 kb)
Additional file 3: Figure S3. Morphological analysis of the Keio collection reveals correlations between cell width and length standard deviation. Contours from cells from each Keio deletion strain were extracted from images acquired from the NBRP repository and used to compute the length and mean width along the cell midline for each cell. The standard deviation of cell length for each strain represents the natural variation in length due to progression through the cell cycle. As expected based on the correlation of mean width and length (Fig. 4a), mean width was correlated with length standard deviation. White circles and error bars were obtained by binning strains by mean width; blue lines are the fit to binned averages. *R* is Pearson’s correlation coefficient; *p*-value was computed with Student’s *t*-test. (PDF 112 kb)
Additional file 4: Figure S4. Morphological analysis of the Keio collection reveals correlations between cell width and intracellular width variability. Contours from cells from each Keio deletion strain were extracted from images acquired from the NBRP repository and used to compute the mean width and width profile across each cell. For each cell, we then computed the standard deviation of the width profile divided by the mean width to obtain the intracellular width variability. White circles and error bars were obtained by binning strains by mean width; blue lines are the fit to binned averages. *R* is Pearson’s correlation coefficient; *p*-value was computed with Student’s *t*-test. (PDF 111 kb)
Additional file 5: Figure S5. Scaled PCA avoids emphasis on large cell length variation. PCA of the correlation matrix attributes greater variation to width, bending, and tapering modes than unscaled PCA (Fig. 4e–h). (A) Representations of the PCA modes around the mean shape. Mode 1 represents tip morphology. Modes 2–4 represent bending, widening, and tapering, respectively. (B) Scatter plots of the proportion of modes 1–4 and mean cell width of each strain demonstrate that width is correlated with variation represented by each of modes 1–4. (PDF 685 kb)
Additional file 6: Table S1. Conditions in chemical genomics screen from [29] that exhibit negative correlation between mean cell width and S-score with *p*-value less than 0.000154 (Bonferroni multiple-hypothesis correction to *p* < 0.05 across 324 conditions; see Methods). **Table S2.** Conditions in chemical genomics screen from [29] that exhibit positive correlation between mean cell width and S-score with *p*-value less than 0.000154 (Bonferroni multiple-hypothesis correction to *p* < 0.05 across 324 conditions; see Methods). **Table S3.** Pairs of COGs and conditions in chemical genomics screen from [29] that exhibit correlations between mean cell width and S-scores with *p*-value less than 0.000154 (Bonferroni multiple-hypothesis correction to *p* < 0.05 across 324 conditions; see Methods). *: description from [29] and generously provided by Athanasios Typas. **Table S4.** Pairs of COGs and conditions in chemical genomics screen from [29] that exhibit correlations between mean cell length and S-scores with *p*-value less than 0.000154 (Bonferroni multiple-hypothesis correction to *p* < 0.05 across 324 conditions; see Methods). *: description from [29] and generously provided by Athanasios Typas. (DOCX 101 kb)
