# Supplementary figures and images for: Rapid, precise quantification of bacterial cellular dimensions across a genomic-scale knockout library
Source: BMC Biol. 2017 Feb 21;15:17. doi: 10.1186/s12915-017-0348-8 (PMC5320674; doi:10.1186/s12915-017-0348-8)

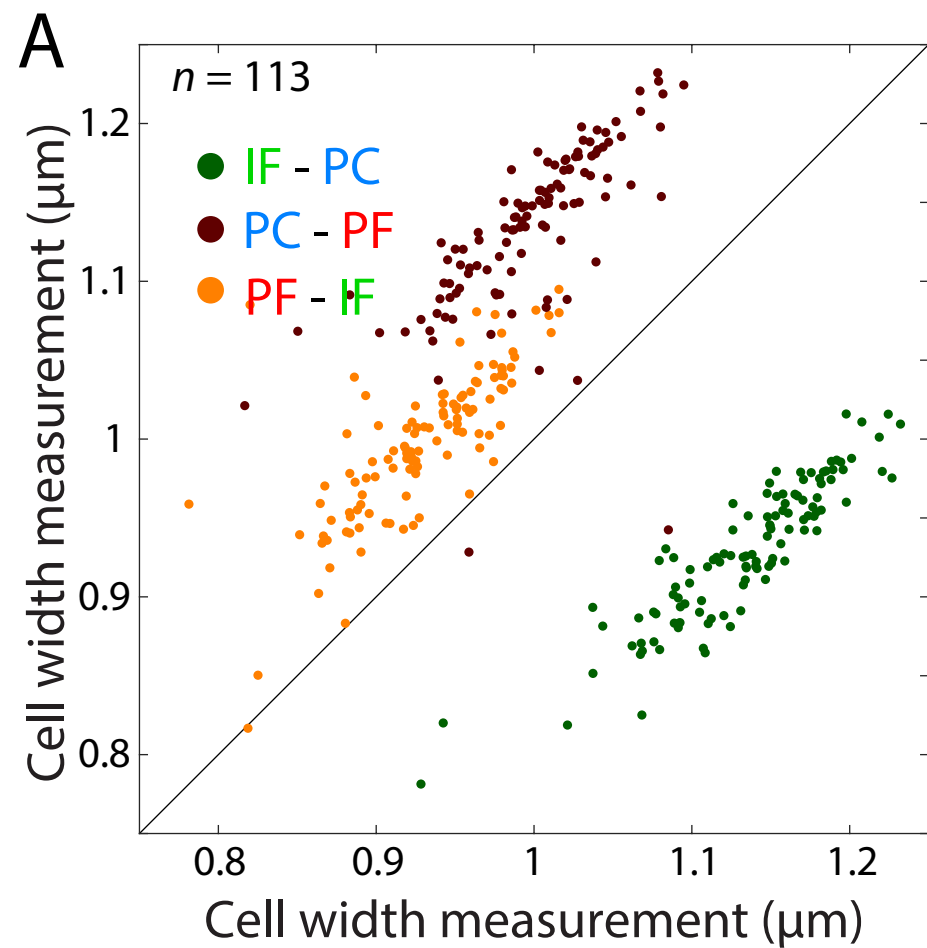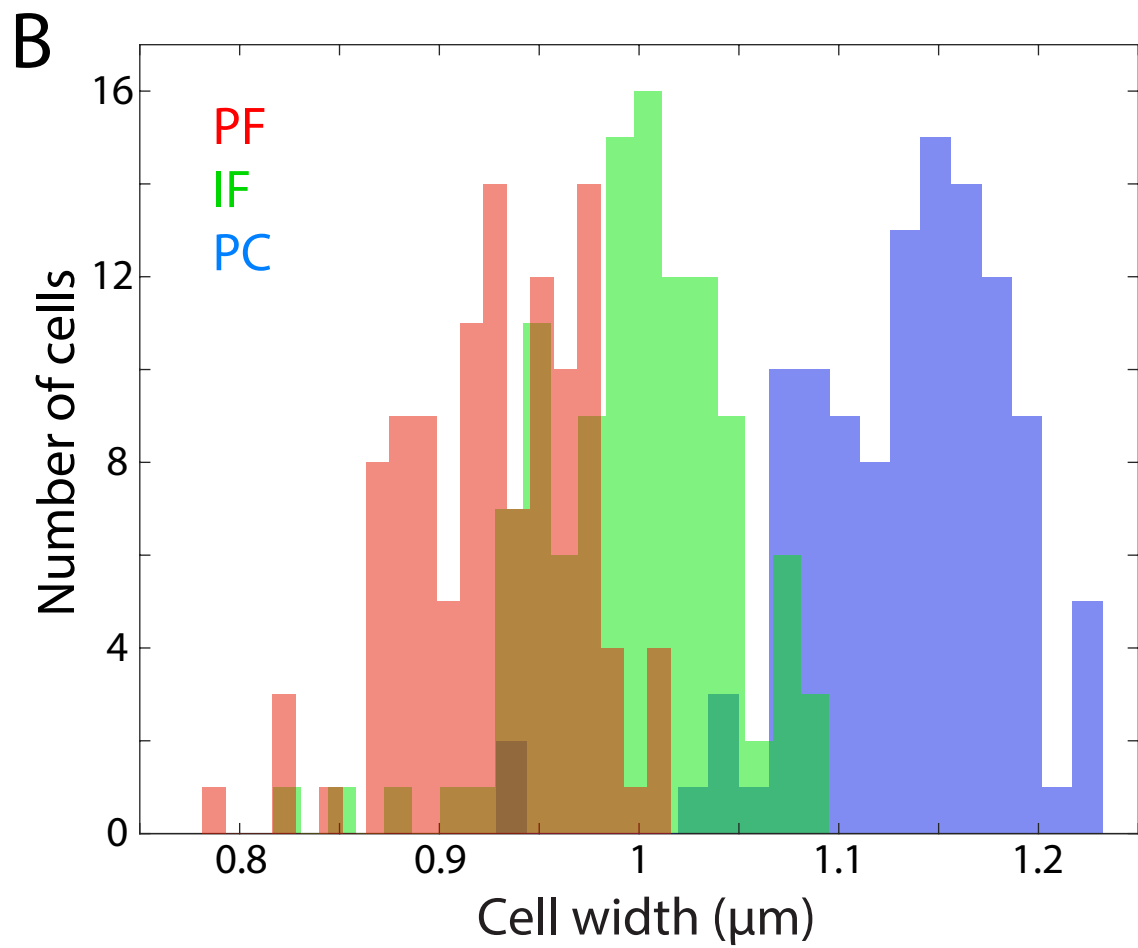

Supplement: Additional file 1: Figure S1. — Width measurements were similarly consistent as length and area across imaging modalities, despite small differences. (A) Differences in width measurements among imaging modalities displayed the same behaviors as length (Fig. 1f) and area (Fig. 1g) across a wide range of cell widths. In the legend, the first and second modality for each color correspond to the measurements along the y- and x-axes, respectively. Black line is y = x. (B) Histograms of cell widths in (A) as measured by each imaging modality. (PDF 132 kb) [file 12915_2017_348_MOESM1_ESM.pdf]

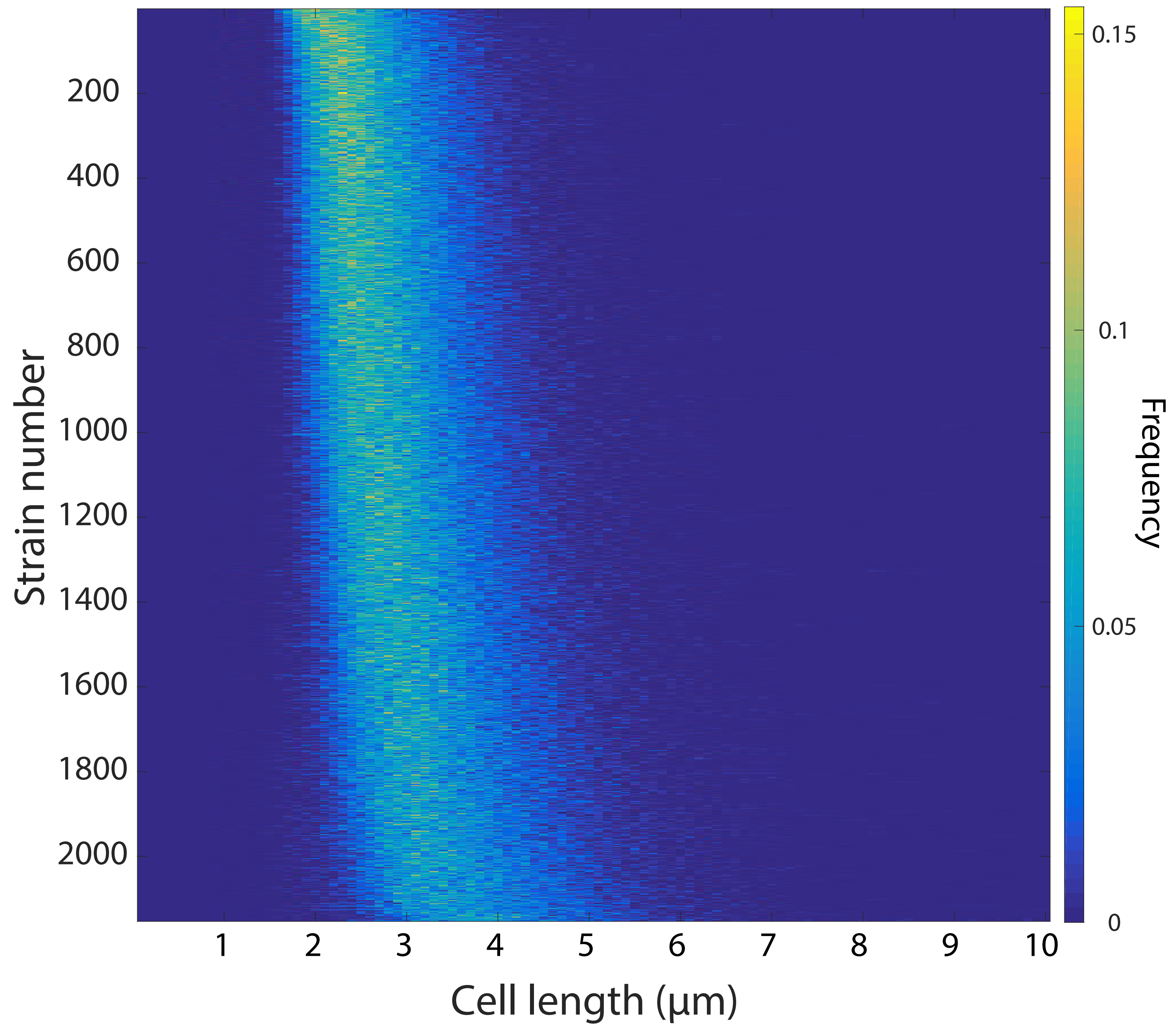

Supplement: Additional file 2: Figure S2. — For all strains used for PCA, the analyzed population had a broad distribution of cell lengths. (PDF 412 kb) [file 12915_2017_348_MOESM2_ESM.pdf]

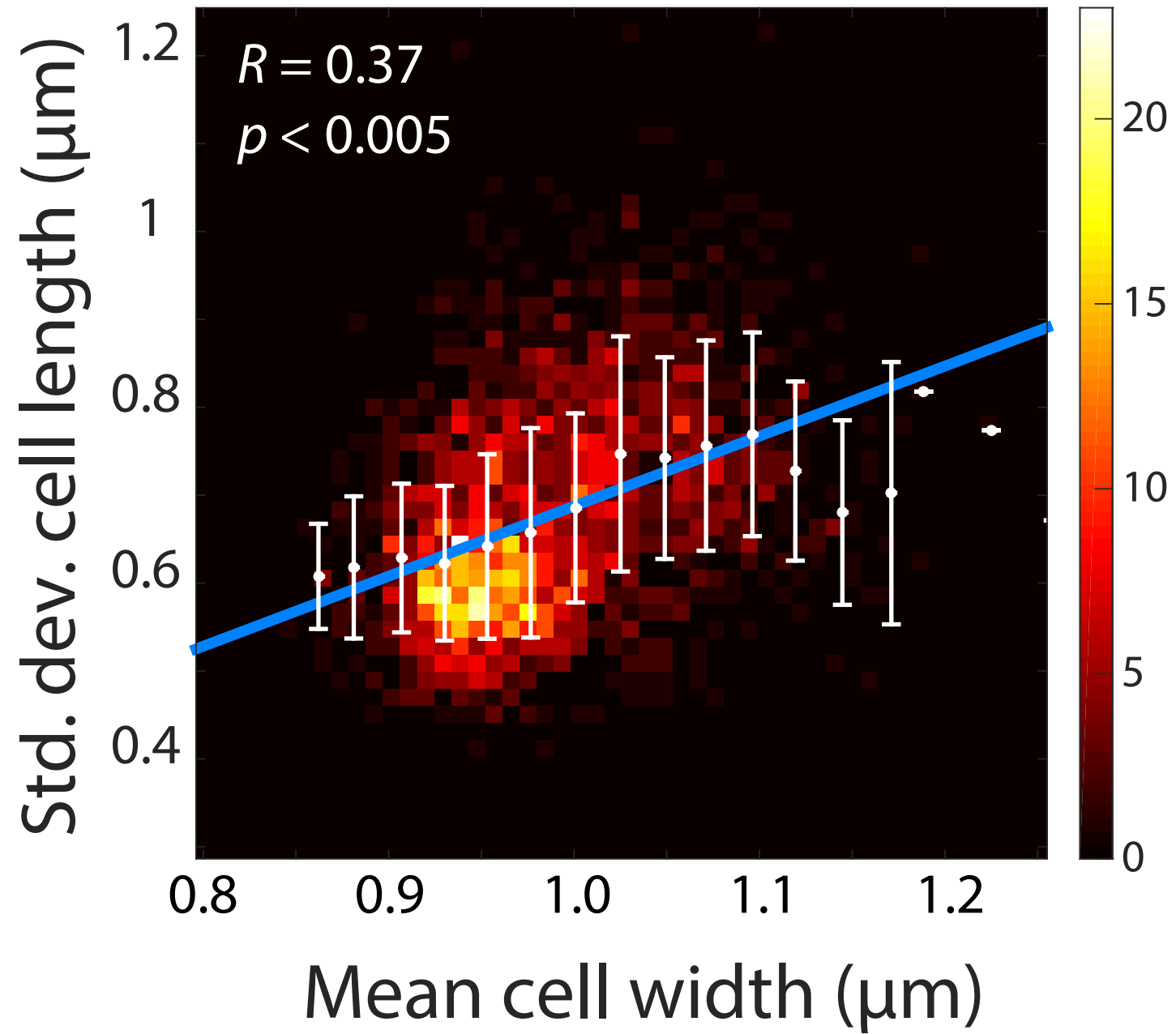

Supplement: Additional file 3: Figure S3. — Morphological analysis of the Keio collection reveals correlations between cell width and length standard deviation. Contours from cells from each Keio deletion strain were extracted from images acquired from the NBRP repository and used to compute the length and mean width along the cell midline for each cell. The standard deviation of cell length for each strain represents the natural variation in length due to progression through the cell cycle. As expected based on the correlation of mean width and length (Fig. 4a), mean width was correlated with length standard deviation. White circles and error bars were obtained by binning strains by mean width; blue lines are the fit to binned averages. R is Pearson’s correlation coefficient; p-value was computed with Student’s t-test. (PDF 112 kb) [file 12915_2017_348_MOESM3_ESM.pdf]

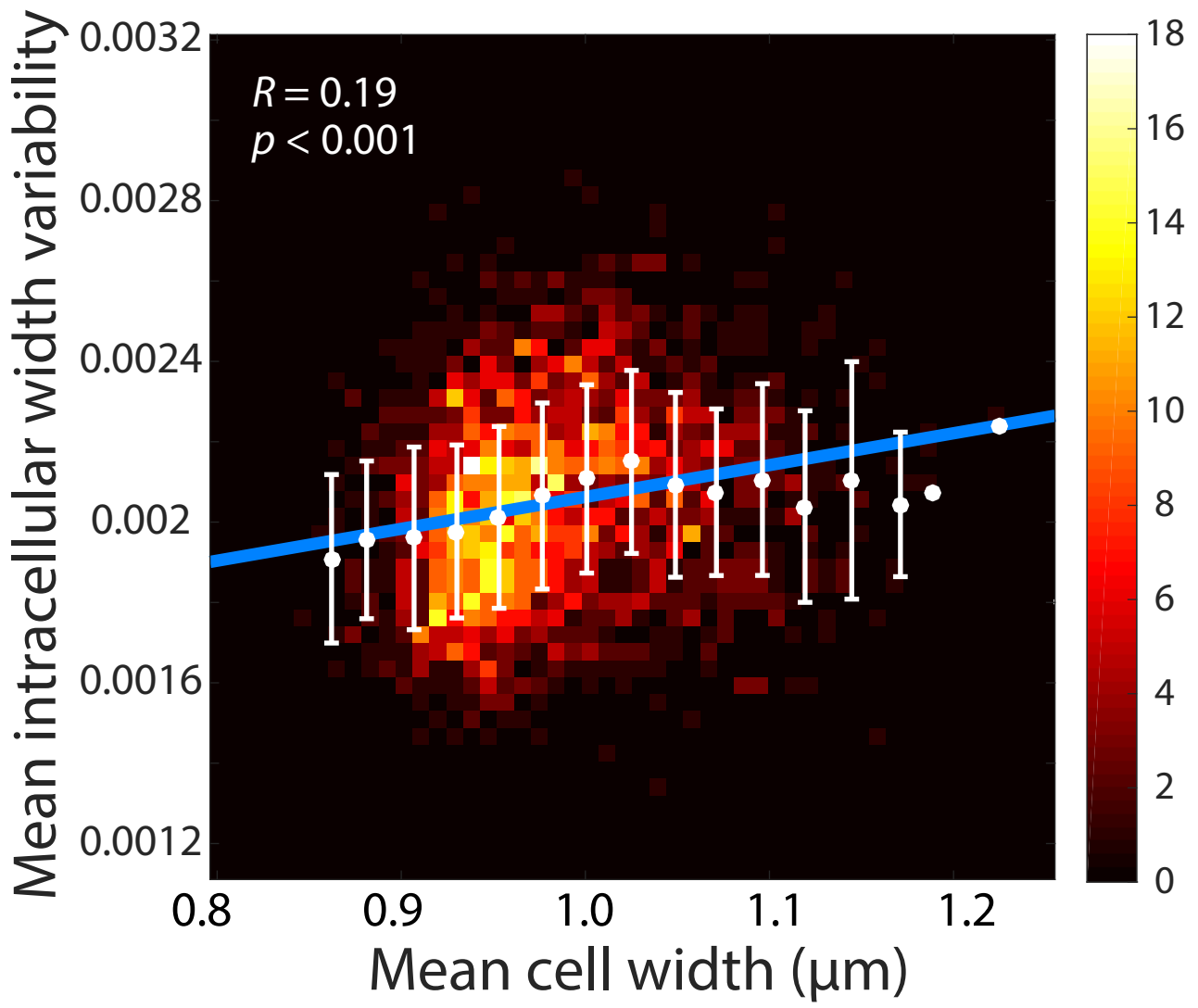

Supplement: Additional file 4: Figure S4. — Morphological analysis of the Keio collection reveals correlations between cell width and intracellular width variability. Contours from cells from each Keio deletion strain were extracted from images acquired from the NBRP repository and used to compute the mean width and width profile across each cell. For each cell, we then computed the standard deviation of the width profile divided by the mean width to obtain the intracellular width variability. White circles and error bars were obtained by binning strains by mean width; blue lines are the fit to binned averages. R is Pearson’s correlation coefficient; p-value was computed with Student’s t-test. (PDF 111 kb) [file 12915_2017_348_MOESM4_ESM.pdf]
