# Supplementary material for: Rapid, precise quantification of bacterial cellular dimensions across a genomic-scale knockout library
Source: BMC Biol. 2017 Feb 21;15:17. doi: 10.1186/s12915-017-0348-8 (PMC5320674; doi:10.1186/s12915-017-0348-8)
Supplement: Additional file 5: Figure S5. — Scaled PCA avoids emphasis on large cell length variation. PCA of the correlation matrix attributes greater variation to width, bending, and tapering modes than unscaled PCA (Fig. 4e–h). (A) Representations of the PCA modes around the mean shape. Mode 1 represents tip morphology. Modes 2–4 represent bending, widening, and tapering, respectively. (B) Scatter plots of the proportion of modes 1–4 and mean cell width of each strain demonstrate that width is correlated with variation represented by each of modes 1–4. (PDF 685 kb) [file 12915_2017_348_MOESM5_ESM.pdf]

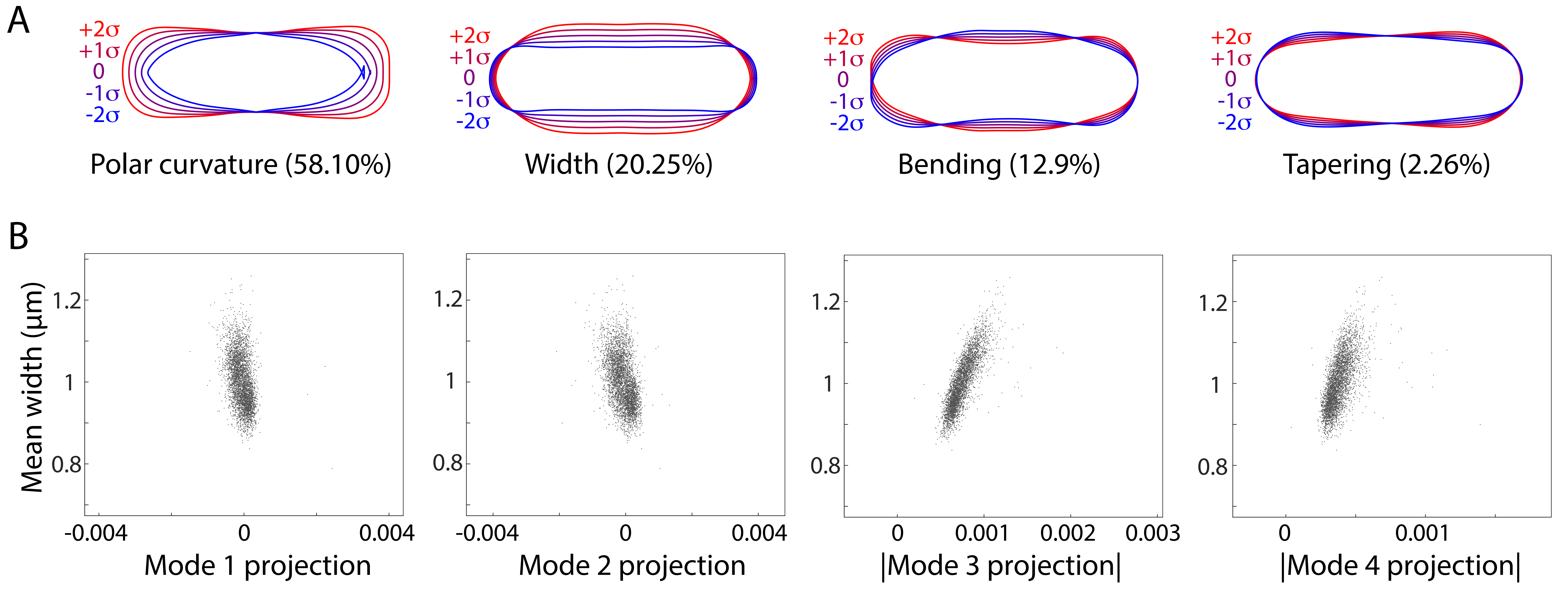
A
+2σ
+1σ
0
-1σ
-2σ
Polar curvature (58.10%)
Width (20.25%)
Bending (12.9%)
Tapering (2.26%)
B
Mean width (μm)
1.2
1
0.8
-0.004
0
0.004
Mode 1 projection
Mode 2 projection
0.001
0.002
0.003
|Mode 3 projection|
|Mode 4 projection|
