# Supplementary material for: Rapid, precise quantification of bacterial cellular dimensions across a genomic-scale knockout library
Source: BMC Biol. 2017 Feb 21;15:17. doi: 10.1186/s12915-017-0348-8 (PMC5320674; doi:10.1186/s12915-017-0348-8)
Supplement: Additional file 6: Table S1. — Conditions in chemical genomics screen from [29] that exhibit negative correlation between mean cell width and S-score with p-value less than 0.000154 (Bonferroni multiple-hypothesis correction to p < 0.05 across 324 conditions; see Methods). Table S2. Conditions in chemical genomics screen from [29] that exhibit positive correlation between mean cell width and S-score with p-value less than 0.000154 (Bonferroni multiple-hypothesis correction to p < 0.05 across 324 conditions; see Methods). Table S3. Pairs of COGs and conditions in chemical genomics screen from [29] that exhibit correlations between mean cell width and S-scores with p-value less than 0.000154 (Bonferroni multiple-hypothesis correction to p < 0.05 across 324 conditions; see Methods). *: description from [29] and generously provided by Athanasios Typas. Table S4. Pairs of COGs and conditions in chemical genomics screen from [29] that exhibit correlations between mean cell length and S-scores with p-value less than 0.000154 (Bonferroni multiple-hypothesis correction to p < 0.05 across 324 conditions; see Methods). *: description from [29] and generously provided by Athanasios Typas. (DOCX 101 kb) [file 12915_2017_348_MOESM6_ESM.docx]

**Supplemental Information for “Rapid, precise quantification of bacterial cellular dimensions across a genomic-scale knockout library”**

Tristan Ursell^1,2,*^, Timothy K. Lee^1,*^, Daisuke Shiomi^4,5^, Handuo Shi^1^, Carolina Tropini^3,9^, Russell D. Monds^1,†^, Alexandre Colavin^3^, Gabriel Billings^6^, Ilina Bhaya-Grossman^1^, Michael Broxton^7^, Bevan Emma Huang^8^, Hironori Niki^4^, Kerwyn Casey Huang^1,9,^^

^1^Department of Bioengineering, Stanford University, Stanford, CA 94305

^2^Department of Physics, University of Oregon, Eugene, OR 97403

^3^Biophysics Program, Stanford University School of Medicine, Stanford, CA 94305

^4^National Institute of Genetics, Shizuoka, Japan

^5^Department of Life Science, Rikkyo University, Tokyo, Japan

^6^Department of Physics, Stanford University, Stanford, CA 94305

^7^Department of Computer Science, Stanford University, Stanford, CA 94305

^8^Janssen Research and Development, Menlo Park, CA 94025

^9^Department of Microbiology and Immunology, Stanford University School of Medicine, Stanford, CA 94305

^*^These authors contributed equally.

^^^Correspondence: [kchuang@stanford.edu](mailto:kchuang@stanford.edu)

^†^Current address: Synthetic Genomics Inc., La Jolla, CA 92037

**Supplementary Information**

**Supplementary Tables**

**Table S1: Conditions in chemical genomics screen from (29) that exhibit negative correlation between mean cell width and S-score with *p­­*-value less than 0.000154 (Bonferroni multiple-hypothesis correction to *p*<0.05 across 324 conditions; see Methods).**

| **Condition** | **Pearson’s correlation** | ***p*-value** |
| --- | --- | --- |
| 0.5 µg/mL A22 | -0.104 | <10^-6^ |
| 2.0 µg/mL cefaclor | -0.084 | 0.000003 |
| 300 µg/mL bacitracin | -0.082 | 0.000004 |
| 0.5 µg/mL verapamil | -0.076 | 0.000022 |
| 0.1 µg/mL verapamil | -0.075 | 0.000028 |
| 16 µg/mL cycloserine D | -0.071 | 0.000069 |
| 0.1 µg/mL erythromycin | -0.070 | 0.000092 |
| 1.0% taurocholate | -0.070 | 0.000096 |

**Table S2: Conditions in chemical genomics screen from (29) that exhibit positive correlation between mean cell width and S-score with *p­­*-value less than 0.000154 (Bonferroni multiple-hypothesis correction to *p*<0.05 across 324 conditions; see Methods).**

| **Condition** | **Pearson’s correlation** | ***p*-value** |
| --- | --- | --- |
| 0.1 µg/mL nitrofurantoin | 0.104 | <10^-6^ |
| 1.0 µg/mL spiramycin | 0.098 | <10^-6^ |
| 0.1 µg/mL streptonigrin | 0.091 | <10^-6^ |
| 40 µg/mL oxacillin | 0.088 | 0.000001 |
| 0.5 µg/mL CCCP | 0.087 | 0.000001 |
| 0.1 µg/mL PMS | 0.087 | 0.000001 |
| 0.1 µg/mL CCCP | 0.083 | 0.000004 |
| 2.0 µg/mL CCCP | 0.081 | 0.000006 |
| 0.1 µg/mL mitomycin C | 0.079 | 0.00001 |
| 10 µg/mL theophylline | 0.077 | 0.000016 |
| 5 µg/mL oxacillin | 0.077 | 0.000017 |
| 100 µg/mL norepinephrine | 0.076 | 0.000019 |
| 0.1 µg/mL high nickel | 0.076 | 0.000021 |
| 0.002 µg/mL levofloxacin | 0.075 | 0.000027 |
| 100 µg/mL theophylline | 0.075 | 0.000028 |
| 0.02 µg/mL PMS | 0.072 | 0.000053 |
| 10 µg/mL pyocyanin | 0.072 | 0.000061 |
| 0.5 µg/mL oxacillin | 0.071 | 0.000075 |
| 2.0 µg/mL nitrofurantoin | 0.070 | 0.000081 |
| 0.05 µg/mL PMS | 0.070 | 0.000087 |
| 5 µg/mL thiolactomycin | 0.069 | 0.000119 |
| 0.02 µg/mL Chir090 | 0.068 | 0.000136 |

**Table S3: Pairs of COGs and conditions in chemical genomics screen from (29) that exhibit correlations between mean cell width and S-scores with *p­­*-value less than 0.000154 (Bonferroni multiple-hypothesis correction to *p*<0.05 across 324 conditions; see Methods). *:** **description from (29) and generously provided by Athanasios Typas.**

| **COG class** | **COG description** | **# genes in COG** | **Condition** | **Condition description^*^** | **Pearson’s correlation** | ***p*-value** |
| --- | --- | --- | --- | --- | --- | --- |
| C | Energy production and conversion | 290 | 0.5 µg/mL amoxicillin | β-lactam | 0.266 | 0.000026 |
| C | Energy production and conversion | 290 | 3.0 µg/mL tunicamycin | Cell-wall biosynthesis inhibitor | 0.251 | 0.000068 |
| C | Energy production and conversion | 290 | 0.1 µg/mL verapamil | Ca^2+^-channel inhibitor | -0.242 | 0.00013 |
| C | Energy production and conversion | 290 | 300 µg/mL sulfamethizole | Sulfonamide | -0.266 | 0.000023 |
| D | Cell cycle control, cell division, chromosome partitioning | 34 | 1.0 µg/mL verapamil | Ca^2+^-channel inhibitor | -0.917 | 0.00001 |
| F | Nucleotide transport and metabolism | 87 | 0.2 µg/mL minocycline | Tetracycline protein synthesis inhibitor | 0.472 | 0.000082 |
| S | Function unknown | 918 | Low Fe | Metal | -0.149 | 0.000067 |
| S | Function unknown | 918 | 300 µg/mL sulfamethizole | Sulfonamide | -0.146 | 0.000095 |
| T | Signal transduction mechanisms | 155 | 0.5 µg/mL A22 | S-benzylisothiourea | -0.376 | 0.000007 |
| T | Signal transduction mechanisms | 155 | 2.0 µg/mL cefaclor | β-lactam | -0.341 | 0.000051 |

**Table S4: Pairs of COGs and conditions in chemical genomics screen from (29) that exhibit correlations between mean cell length and S-scores with *p­­*-value less than 0.000154 (Bonferroni multiple-hypothesis correction to *p*<0.05 across 324 conditions; see Methods). *: description from (29) and generously provided by Athanasios Typas.**

| **COG class** | **COG description** | **# genes in COG** | **Condition** | **Condition description^*^** | **Pearson’s correlation** | ***p*-value** |
| --- | --- | --- | --- | --- | --- | --- |
| E | Amino acid transport and metabolism | 421 | Acetate | Carbon source | -0.209 | 0.000095 |
| E | Amino acid transport and metabolism | 421 | Glucosamine | Carbon source | -0.205 | 0.000124 |
| E | Amino acid transport and metabolism | 421 | Glycerol | Carbon source | -0.214 | 0.000064 |
| E | Amino acid transport and metabolism | 421 | Maltose | Carbon source | -0.224 | 0.000026 |
| K | Transcription | 319 | 0.2 µg/mL tobramycin | Aminoglycoside | 0.245 | 0.000062 |
| O | Post-translational modification, protein turnover, chaperones | 138 | 0.05 µg/mL gentamycin | Aminoglycoside | 0.372 | 0.000105 |
| S | Function unknown | 918 | 3.0 µg/mL chlorpromazine | Antipyschotic | 0.151 | 0.000053 |
| S | Function unknown | 918 | 0.1% deoxycholate | Detergent | -0.147 | 0.000077 |
| S | Function unknown | 918 | 0.05 µg/mL triclosan | Phenol | 0.142 | 0.000144 |
